# Dose Titration of Solid Dosage Forms via FDM 3D-Printed Mini-Tablets

**DOI:** 10.3390/pharmaceutics14112305

**Published:** 2022-10-27

**Authors:** Guluzar Gorkem Buyukgoz, Christopher G. Kossor, Shen Ji, Murat Guvendiren, Rajesh N. Davé

**Affiliations:** 1 New Jersey Center for Engineered Particulates (NJCEP), New Jersey Institute of Technology, Newark, NJ 07102, USA; 2 Otto H. York Department of Chemical and Materials Engineering, New Jersey Institute of Technology, Newark, NJ 07102, USA

**Keywords:** FDM 3D-printing, mini-tablets, low-dose titration, personalized medicine, split tablets, high and low drug concentrations

## Abstract

The robustness of 3D-printed mini-tablets as a platform to administer milligram dosages, intended for age-specific therapy, without the need of tablet splitting while maintaining similar release profiles, was investigated. Griseofulvin, as a model poorly water-soluble drug, and hydroxypropyl cellulose along with Kollicoat Protect as polymers were used to prepare filaments at 1–20% drug concentrations via hot-melt extrusion (HME). Higher drug concentrations served for testing the feasibility of a reduced number of mini-tablets to be administered. A reliable dose titration in the range 0.19–3.91 mg at a high accuracy (R^2^ of 0.999) was achieved through composite unit (multi-unit) mini-tablets. All mini-tablets produced had excellent content uniformity and their label claim values were within the acceptable range, proving that HME processing followed by 3D printing promotes content uniformity even for mini-tablets containing low drug doses (0.19 mg). Remarkably, the proposed approach allowed achieving similar drug release profiles via composite unit mini-tablets as well as single mini-tablets at high drug concentrations. In contrast, split tablets demonstrated different release behaviors, attributed to their size and shape differences. Overall, the distinct advantages of mini-tablets to provide dose flexibility while maintaining similar release profiles was demonstrated.

## 1. Introduction

Many pediatric formulations require dose manipulation [1,2,3,4,5,6] to either administer prescribed doses per age/body weight or to minimize swallowing issues. Splitting a large tablet into smaller pieces [7,8,9,10,11] is the most prevalent and simplest practice for manipulating solid dosage forms. However, the respective fragments formed may not meet the intended dose owing to the possible variation in weight, content uniformity [7,9,12,13,14], and the effect on drug dissolution, as well as subsequent metabolism [12,15]. Resulting inconsistencies in the delivered drug amount could prove detrimental for patients who are at risk when the drug has a narrow therapeutic index [12,16]. As an alternative, liquid dosage forms have been used for pediatric patients, but accurate dosing remains challenging due to issues related to failure of following dosing instructions, dosage form stability, and possible contamination during administration [17,18,19,20,21]. Nevertheless, such issues drive the need to develop better solutions for patient-tailored, age-specific formulations [3,4].

Oral multi-particulate drug delivery systems, such as nano/microparticles, granules, pellets, and mini-tablets, could be an alternative solution for age-specific formulations by tailoring their total counts as a function of patient age [22,23]. Amongst the multi-particulate systems, mini-tablets offer attractive advantages, i.e., mechanical properties, constant specific surface area, smooth outer surface, and reliable size and shape [22,24,25]. Therefore, mini-tablets have emerged as one such solution and have enormous potential as an age-specific drug therapy [2,21,26,27,28,29]. Typically, mini-tablets are defined by their smaller size with about 3–5 mm in diameter [30,31,32,33,34]. Their relatively small size could be beneficial to mitigate swallowing issues and offer a high level of patient compliance through a single or composite (multi-unit) tablet administration [21,26,34,35,36]. Particularly, multi-unit mini-tablets loaded with low drug concentrations enable higher flexibility for low-dose drug delivery [21]. Furthermore, they are less sensitive to external factors, unlike liquid forms, and have a greater dose accuracy to potentially prevent failure in attaining therapeutic concentration, which can occur with fragmented tablets or subdivided adult tablets [12,17,26]. Consequently, the focus of this work is the examination of these distinct advantages of mini-tablets to provide dose flexibility for age-specific patients.

Traditionally, mini-tablets are formed via compression and their manufacturing steps are similar to standard size tablets by mixing and compressing via conventional tablet presses equipped with multiple punches [22,37]. The uniformity and consistency in such small-sized products naturally requires additional efforts in the formulation development and processing because of the requirements such as: an upper limit on the particle size to avoid clogging of the die opening [38], excellent flow properties to meet the consistency in die filling [22,37,39], and strict control of the tablet tool alignment against the high die-wall friction [34,37]. The aforementioned challenges with traditional manufacturing of mini-tablets form a motivation for exploring alternate approaches. The emerging field of 3D printing technology offers new and exciting opportunities for overcoming some of the aforementioned limitations. It can provide the manufacturing flexibility through printing complex product shapes [40] and including uniform particles at a small scale [41,42,43,44,45,46,47]. Fused deposition modeling (FDM) based 3D printing [47,48,49,50,51,52,53] is appealing due to its rapid manufacturing speed unlike SPHRINT (g h^−1^) and quick post-printing processes in contrast to post-curing after stereolithography (SLA) and powder removal after selective laser sintering (SLS) [41,44,45,46]. In addition, FDM 3D printing appears to eliminate challenges associated with compressed powder tableting since the starting material is a thermoplastic solid filament [54,55,56,57]. The manufacturing of filaments is performed via hot-melt extrusion (HME) which avoids all powder-related problems and offers the additional benefit of potentially enhancing content uniformity through intense mixing actions [58,59,60]. Hence, the HME process coupled with 3D printing would promote dosing reliability; see for example, the reported results of improved content uniformity [61,62,63,64]. In FDM 3D printing, the intended dose is precisely deposited through successive layers of thermoplastic filaments [62,65] with high precision through digital control.

Due to its potential for flexible dosing, the FDM 3D printing platform has been actively researched for dose titration [66,67,68], preparing appealing designs for children [61], and for low-dose formulations, i.e., <5.0 mg [68,69]. Although these examples suggest obvious applications of FDM 3D printing for addressing the needs of pediatric patients, interestingly, its use in printing mini-sized tablets has not been well explored [70]. Further, the reported examples of FDM 3D-printed mini-tablets did not investigate their application to dose titration [71], hence, did not take full advantage of the possibilities offered by mini-tablets. The present study intends to investigate the capability of 3D-printed mini-tablets to deliver varying doses to pediatric patients without compromising the intended therapeutic effect by adversely impacting the drug dissolution or drug content uniformity.

Towards that objective, 3D-printed miniature-sized tablets with a set diameter of 3 mm containing a low drug concentration of griseofulvin, a model biopharmaceutical classification system (BCS) class II drug, as single or multi-units are examined for their ability to administer the prescribed dose, including low-dose drug titration. The focus is not only achieving reliable and flexible dosing, but also obtaining similar drug release profiles via using multi-unit mini-tablets, even as tablet count varies. Hydroxypropyl cellulose (HPC) is used as polymer since it offers the mechanical resilience required for the printability of the filaments [57,62,72,73,74], and its recently demonstrated capability in achieving similar release profiles for varying concentrations of poorly water-soluble drugs through a constant tablet surface area to volume ratio (SA/V) [75]. In addition to low drug concentrations, higher drug concentration filaments are also examined for the purpose of reducing the number of tablets to be administered, albeit limiting the dose titration capability. Finally, printed multi-unit and split tablets are compared to gain insight into the robustness of the 3D-printed mini dosage forms. Toward these goals, the following design options are examined:

- Single and multi-unit mini-tablets, i.e., 1, 5, 10, 15, 20 count(s), to test content uniformity at a low dose, i.e., 1 wt%, and to evaluate corresponding dissolution profiles to assess their similarity.
- Split tablets, i.e., full, half, and quarter sizes, to compare with multi-unit mini-tablets along with testing content uniformity and dissolution.
- Single unit mini-tablets formed using filaments of 10 and 20 wt% drug concentrations for testing feasibility of reducing the number of tablets to be administered.

## 2. Materials and Methods

### 2.1. Materials

As received griseofulvin (GF) (Letco Medical, Decatur, AL, USA) with a primary particle size of ~11 µm was selected as a model biopharmaceutics classification system (BCS) class II drug. Hydroxypropyl cellulose (HPC, SL grade) was donated by Nisso America Inc. (New York, NY, USA). Kollicoat^®^ Protect (KP) was donated by BASF (Tarrytown, NY, USA). KP is composed of polyvinyl alcohol-polyethylene glycol graft copolymer, polyvinyl alcohol, and fumed silica. It is a readily soluble polymer in water and known to improve protection against moisture [76]. Additionally, the hydroxyl groups of HPC and KP can potentially form hydrogen bonds with the carbonyl groups of GF [77,78], which could improve the homogeneity of the matrix [79], leading to filaments with desirable mechanical properties that are suitable for printing. Sodium dodecyl sulfate (SDS) (Sigma-Aldrich, Saint Louis, MO, USA) was used as a solvent.

### 2.2. Manufacturing of Filaments

Table 1 presents the composition of the powder blends used for the manufacturing of filaments. To mix the powder blends, a high-intensity vibrational mixer (LabRAM, Resodyn Acoustic Mixers, Inc., Butte, MT, USA) was used at a frequency of 61 Hz with an acceleration of 75 G for 5 min. The powder blend was extruded through an 11 mm diameter co-rotating twin-screw extruder (Thermo Fisher Scientific Inc., Waltham, MA, USA) with the processing temperatures and screw speed presented in Table 1. A round-shaped die with a 2 mm opening was used to extrude the molten blend. The extrusion temperature and screw speed were optimized for the formulations at 1.0 and 10.0 wt% drug concentrations; see Table 1. Briefly, the torque and pressure values were considered to ensure proper instrument safety and to provide proper mixing [32,79,80]. Additionally, the thermal stability and softening of materials were considered to achieve filaments with mechanical resilience for printing. Thus, extrusion at 150 °C resulted in an acceptable processing torque (3.4–3.7 Nm) within safe HME processing and provided uniform, consistent filaments. Higher drug concentrations in the formulation require higher energy input to soften the material when a drug has greater softening needs than that of a polymer [75]. Therefore, the extrusion temperature for the formulation containing 20.0 wt% drug concentration was increased to 155 °C.

### 2.3. FDM 3D Printing and Tablet Morphology

**Figure 1:**
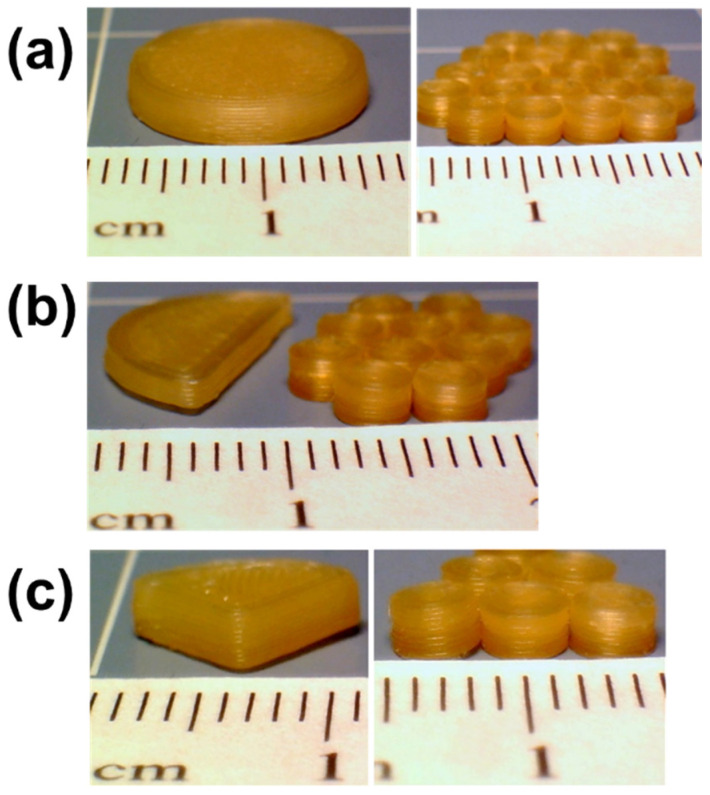
Digital images of (**a**) full tablet vs. twenty units mini-tablets, (**b**) half tablet vs. ten units mini-tablets, and (**c**) quarter tablet vs. five units mini-tablets, each sub case contains similar drug amounts (all the tablets were printed using the formulation F1 listed in Table 1).

The intended doses were printed as mini-tablets containing varying drug concentration, split tablets, and multi-unit mini-tablets. The tablets printed with filaments containing 1.0 wt% drug concentrations enabled titrating the dose with small increments, i.e., <1.0 mg. The tablets containing higher drug concentrations aimed to reduce the number of tablets necessary to be administered. The printed subdivided tablets were examined to compare multi-unit tablets containing similar drug amounts, see Figure 1 for digital images of the printed tablets. The details of tablet properties, dimensions, and count(s) are shown in Table 2. All tablet designs were created using Autodesk^®^ Fusion 360 Ultimate (Autodesk 3D design software, Version 2.0. 14336; Boston, MA, USA) and recorded as an STL file. The designs were sliced using FlashPrint software (Flashforge^®^, Version 3.18.0; Jinhua, China) and printed using FDM 3D printer (Flashforge^®^, Creator Pro 3D, 2016, China). The printer nozzle used a 4 mm opening. In all of the print designs, the printing temperature of 180 °C, the print speed of 35 mm/s, the travelling speed of 80 mm/s, 100% infill, and layer height of 0.2 mm were applied. The printing temperature was set to meet the general guidelines that FDM processing requires which is a higher processing temperature than that of HME owing to lacking of high shear [62]. The images of the 3D-printed mini-tablets at 1.0 and 20.0 wt% drug concentrations were acquired to demonstrate the layered structure of the tablets via scanning electron microscopy (JSM-7900F, JEOL Ltd., Peabody, MA, USA).

### 2.4. Thermo-Gravimetric Analysis (TGA)

Any thermal degradation events stemming from thermal processing in FDM 3D printing of HPC, KP and as received GF powders, physical mixtures (PM), and the printed tablets were tested by thermo-gravimetric analysis (TGA) (TGA/DSC1/SF STARe system, Mettler Toledo Inc., Columbus, OH, USA). In a standard ceramic crucible, the samples were heated from 25 to 240 °C at a rate of 10 °C/min and cooled back to 25 °C under a nitrogen flow.

### 2.5. Solid-State Characterization

X-ray diffraction was performed to analyze the solid state of GF after the processing of FDM 3D printing. The tablets were reprinted following the method in Buyukgoz et al. [75] in order to fit onto the XRD sample holder. Diffraction patterns were acquired for the samples using PANalytical (Westborough, MA, USA), scanning for the 2-theta angle within the range of 5–30° (0.01° step). Further, the physical mixtures, filaments, and the printed tablets were examined for physical transformations of the drug using a differential scanning calorimeter (DSC 6000, Perkin Elmer, Inc., Waltham, MA, USA). The samples, 5–8 mg each, were heated in a standard aluminum pan from 25 to 300 °C at a rate of 10 °C/min. Nitrogen was used as a purge gas at a flow rate of 20 mL/min.

### 2.6. Content Uniformity

The variations in tablet size, tablet weight, and drug mass were measured from randomly collected printed mini-tablets. The sample size for the single unit mini-tablets containing low drug concentrations, 1.0 wt%, was *n* = 30 [21,81]. However, the sample size was kept as *n* = 3 for the tablets with the higher drug concentrations, 10.0 and 20.0 wt%, and for the multi-unit (composite) tablets comprised of five, ten, fifteen and twenty count per sample. Content uniformity in multi-unit mini-tablets was assessed to determine the critical number of mini-tablets with acceptable dose variability. The tablet weights and dimensions were recorded. Each sample was dissolved in 7.2 g/L SDS solution while stirred via magnetic bars overnight. The dissolved samples were filtered with a 0.45 µm nylon membrane-type syringe filter (Celltreat scientific products, Pepperell, MA, USA), and analyzed for GF content and uniformity at 297 nm UV absorbance wavelength using a Thermo Scientific Evolution 300 UV–Vis spectrophotometer (Thermo Fisher Scientific Inc., Waltham, MA, USA). The acceptance criteria given in USP<905> [81] were also applied for the assessment of content uniformity testing.

### 2.7. Dissolution

The release behaviors of printed tablets in Table 2 were examined by comparing: (a) 1–20 unit(s) of mini-tablets, (b) composite units vs. split tablets, and (c) mini tablets at varying drug concentrations. Accordingly, the proposed approach of achieving a similar drug release profile from multi-unit tablets, where the total drug amount varies as per tablet count(s), was examined with 1–20 unit(s) of mini-tablets. The resulting release profiles were compared with split tablets and single unit mini-tablets that contained higher drug concentrations. The samples compared were designed to contain similar drug amounts to prevent any bias on the dissolution performance of the tablets arising from the differences in their drug content. For example, the total drug amount in ten mini-tablets containing 1.0 wt% drug was attempted to be kept similar to the drug amount of a single mini-tablet with 10 wt% drug or a half-split large tablet containing 1.0 wt%. The dissolution paddle apparatus (USP II, Sotax, Aesch, Switzerland) was used for testing the release profiles of the individual and multi-unit mini-tablets. The sinkers were used for the multi-units to prevent the tablets from floating around. Deionized water (DI) was used as a dissolution medium [82,83]. Testing the drug release profiles in a large volume of dissolution media can give rise to analytical detection problems particularly when the amount of drug is limited, e.g., in mini-tablets [84]. The samples were added to 500 mL of dissolution medium at 37 °C, where the sink conditions were maintained. The paddle speed at 100 rpm was used to minimize the coning effect and to provide reliable dissolution results, particularly for poorly soluble drugs [84,85]. Aliquots were withdrawn at certain time intervals over 24 h and filtered through a 0.45 µm nylon membrane-type syringe filter (Celltreat scientific products, Pepperell, MA, USA). The filtrate was analyzed for the average percentage of the dissolved GF at 297 nm UV absorbance wavelength using a Thermo Scientific Evolution 300 UV–Vis spectrophotometer (Thermo Fisher Scientific Inc., Waltham, MA, USA). The average percentage of the dissolved drug was plotted as a function of time. Each test was replicated a minimum of three times.

## 3. Results

### 3.1. Mini-Tablet Printing and Tablet Morphology

The mini-sized tablets with cylindrical shapes were successfully printed via FDM 3D printing. The inherently small size of the mini-tablet allowed for printing multiple tablets at a time, potentially improving the consistency between printed units by minimizing the inconsistencies arising from material changeover and tablet collection. The SEM images exhibit a similar layered structure of mini-tablets at 1.0 and 20.0 wt% drug concentrations (see Figure S1, Supplementary Material).

### 3.2. Thermo-Gravimetric Analysis (TGA)

**Figure 2:**
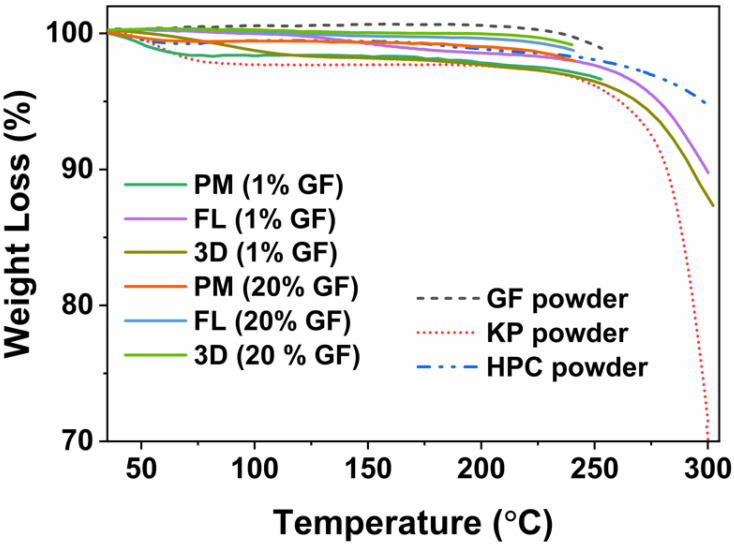
TGA thermograms for GF, HPC and KP powders, physical mixtures (PMs), filaments, and printed tablets at 1.0 and 20.0 wt% drug concentrations.

The outcomes of the thermo-gravimetric analysis (TGA) are presented in Figure 2. The highest weight loss was observed for KP powder, which was less than 2.3% at 100 °C, which could be attributed to the free or bound water [86]. This is in line with Wei et al. [55], reporting that 2–5% weight loss at temperatures up to 100 °C implies the loosely bound moisture. Increasing the temperature did not increase the weight loss (2.4%) much, indicating no possibility of thermal degradation at the printing temperature. The weight loss for all other powder materials and 3D tablets was also less than 2.4%, indicating their thermal stability throughout the mini-tablet printing process.

### 3.3. X-ray Diffraction (XRD)

**Figure 3:**
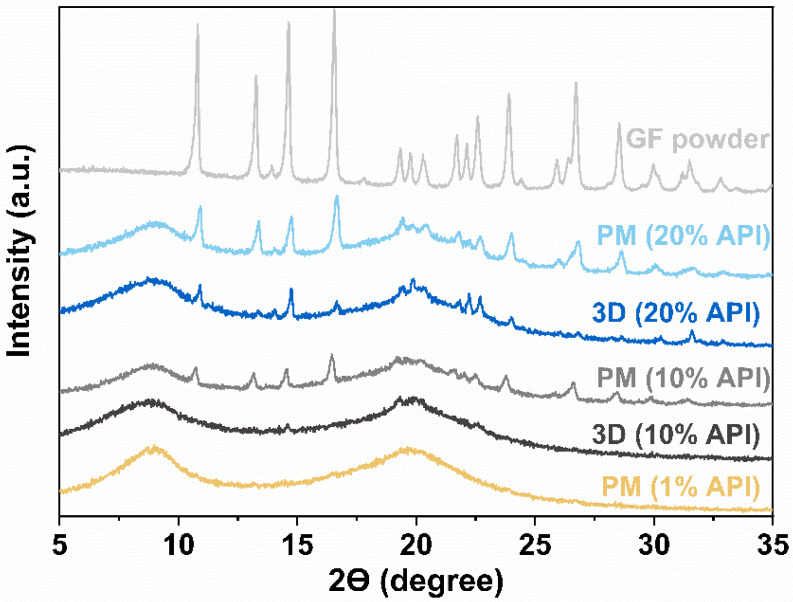
XRD diffractograms of GF powder, physical mixtures (PMs), and printed tablets.

The crystalline state of GF after 3D printing was assessed using XRD on: as received GF, HPC and KP powders, PMs, and printed tablets. The XRD diffractograms, presented in Figure 3, indicate that the polymers are amorphous due to the presence of halo patterns, while GF exhibits the crystallinity due to the presence of its characteristic peaks [87]. The diffractogram of PM for 1 wt% GF lacked the characteristic peaks of GF and exhibited a halo pattern, most likely because the low drug concentration is below the limit of detection for determining GF crystallinity [88]. Further, the PMs with the drug concentrations of 10 and 20 wt% showed similar characteristic peaks of GF and their lower intensity could be attributed to the surface coverage and dilution of GF particles with polymers [83,87]. In the diffractograms of printed tablets containing 20 and 10 wt% GF concentration, the diminished intensities or absences of the characteristic peaks of GF at 13.2° and 16.5° [87] indicate partial miscibility of GF with polymers after heat treatment occurring from 3D printing. The partial miscibility of GF–HPC has also been reported by Rahman et al. [77]. Additionally, these observations were further supported by the DSC results, see Figure S2, Supplementary Material.

### 3.4. Content Uniformity and Dose Titration

#### 3.4.1. Content Uniformity

The variations in drug mass and tablet weight for FDM 3D-printed mini-tablets and split tablets, are shown in Table 3. The dimensions of the single unit mini-tablet are also shown in Table S1, Supplementary Material. As per USP <905> L2 criteria [81], the acceptance value (AV) for *n* = 30 units cannot exceed 25.0. All mini-tablets had excellent content uniformity with AV < 7.9. The relative standard deviation (RSD) values in drug concentrations for all tablets were less than 4.7% and their label claim (LC) values were within the acceptable range of ±25% of the target dose, see Table 3. The enhanced content uniformity is not surprising because the intense mixing effect of HME could produce contently uniform products [58,59,60]. In addition, FDM 3D-printed mini-tablets exhibited closely similar weights most likely due to the capability of the 3D printer to create tablets with precise dimensions, which further facilitates the uniformity in drug content. This observation is in line with the studies performing HME for filament manufacturing and achieving contently uniform miniature tablets [32,70]. Surprisingly, Ayyoubi et al. [89] reported large variations and drug loss of up to 8.5% from FDM 3D-printed mini-tablets. These issues faced during the manufacturing of the filaments via a single-screw extruder and printing at high temperatures caused drug degradation. These results emphasize the importance of optimizing the printing processing parameters and the effectiveness of the twin-screw extruder to achieve good content uniformity. It is important to highlight that the enhanced uniformity achieved through the combination of HME and FDM 3D printing. It could offer an alternative approach to the traditional mini-tablet manufacturing process which requires extra effort in the judicious selection of several processing parameters uniformity (e.g., upper limit on the particle size, flow properties, tool alignments [22,37,39]) as they impact the content uniformity.

#### 3.4.2. Dose Titration

**Figure 4:**
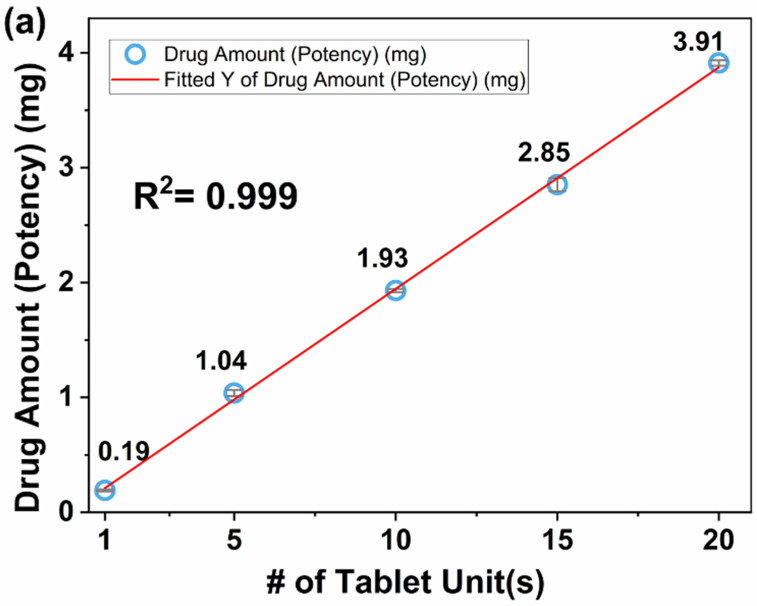

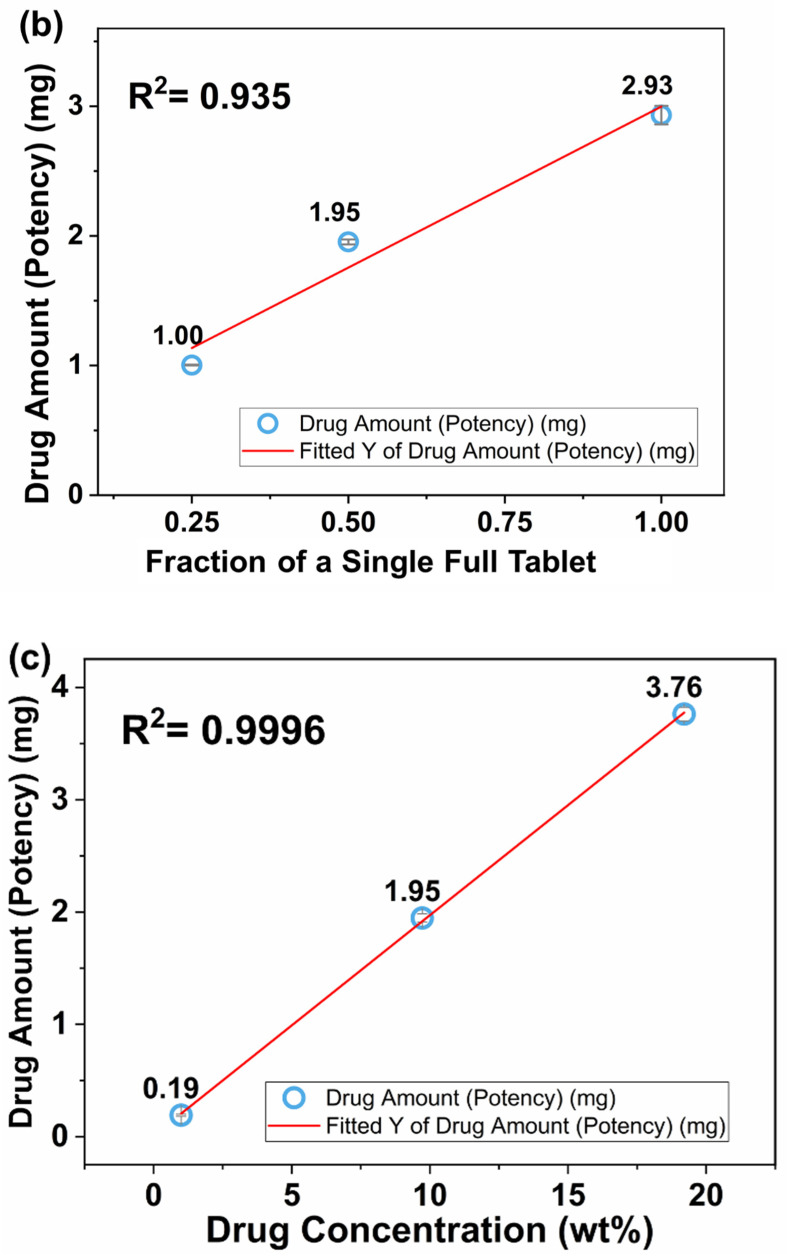
Dose titration via (**a**) multi-unit mini-tablets at 1.0 wt% drug concentration, (**b**) split tablets at 1.0 wt% drug concentration, and (**c**) single unit mini-tablets at 1.0–20.0 wt% drug concentrations.

The single unit mini-tablet produced with the lowest drug concentration filaments enabled dose titration in steps of 0.19 mg to cover the dispensed dose in range of 0.19–3.91 mg using 1–20 counts of mini-tablets. Tablet count(s) versus drug amounts were plotted to evaluate the robustness of the dose titration, see the plots in Figure 4. In Figure 4a, a high accuracy with the R^2^ of 0.999 was observed. Generally, the parameter critical to determining the minimum count(s) of mini-tablets necessary for an acceptable low dose and dosing variability is based on the composite units and not a single mini-tablet [21]. However, the use of 3D printing led to excellent content uniformity of the single unit mini-tablet itself. The linear trend achieved (R^2^ of 0.999) with increasing tablet count(s) confirmed the reliability of the dosage consisting of a single unit mini-tablet, eliminating the need for using a multi-unit mini-tablet to determine the minimum reliable dose.

Rycerz et al. [67] achieved dose titration from specially designed printing patterns. In their novel design, the dispensed dose was adjusted with the printing process parameters or the portions of the printing patterns. It is worth mentioning that the mini-tablets containing ~0.19 mg drug eliminates the need for modifications of the printing parameters and allows dose titration by administering the desired number of tablets.

Tablet fraction(s), i.e., full, half, and quarter, versus drug amounts were plotted to compare the performance of the split tablet in terms of dose titration, see Figure 4b. All printed splits tablets were contently uniform and their LC% values were in the acceptable range (see Table 3). This level of uniformity was expected since the fragmentation issue of split tablets was not a concern because they were precisely printed instead of having to divide them into pieces. However, this did not translate into the enhanced linearity in dose titration, where R^2^ was found 0.935. The reduced linearity may have a negative effect on the dosage reliability. Unlike the multiple mini-tablets, where each has similar final mass, the variation in final mass for the split tablets depends on their final shape and size. The variation in dosage for the split tablets could be explained by: (i) the swelling behavior of HPC, which is expected to be different for various tablet shapes/sizes and (ii) the lesser quality of the equipment used.

Single unit mini-tablets containing higher drug concentrations, see Table 3, were examined in an attempt to reduce the number of tablets to be administered for the intended dose. They showed enhanced uniformity with acceptable LC% values. As previously reported, using filaments of varying drug concentrations is a reliable practice for the dose adjustment [75]. Thus, the resulting uniformity at higher concentrations was expected to be similar to a single unit mini-tablet at low concentration. Indeed, the performance in dose titration for the tablets at higher drug concentrations also showed high linearity with R^2^ of 0.9996, see Figure 4c. Most remarkably, the drug amounts in twenty single mini-tablets containing 1.0 wt% drug concentration, resulted in the dose of 3.91 ± 0.02 mg, whereas one single mini-tablet containing 20.0 wt% drug concentration, resulted in the dose of 3.76 ± 0.06, Table 3. These values are similar, although the higher drug concentration of the filament limited the escalation extent in dose titration. Regardless, the use of higher drug concentration filaments significantly reduces the number of tablets to be administered and could be useful for the age-specific formulations without an appreciable loss of prevision in dose titration. These are the major strengths of the 3D printing approach for producing mini-tablets suitable for age-specific dosages.

### 3.5. Drug Dissolution

**Figure 5:**
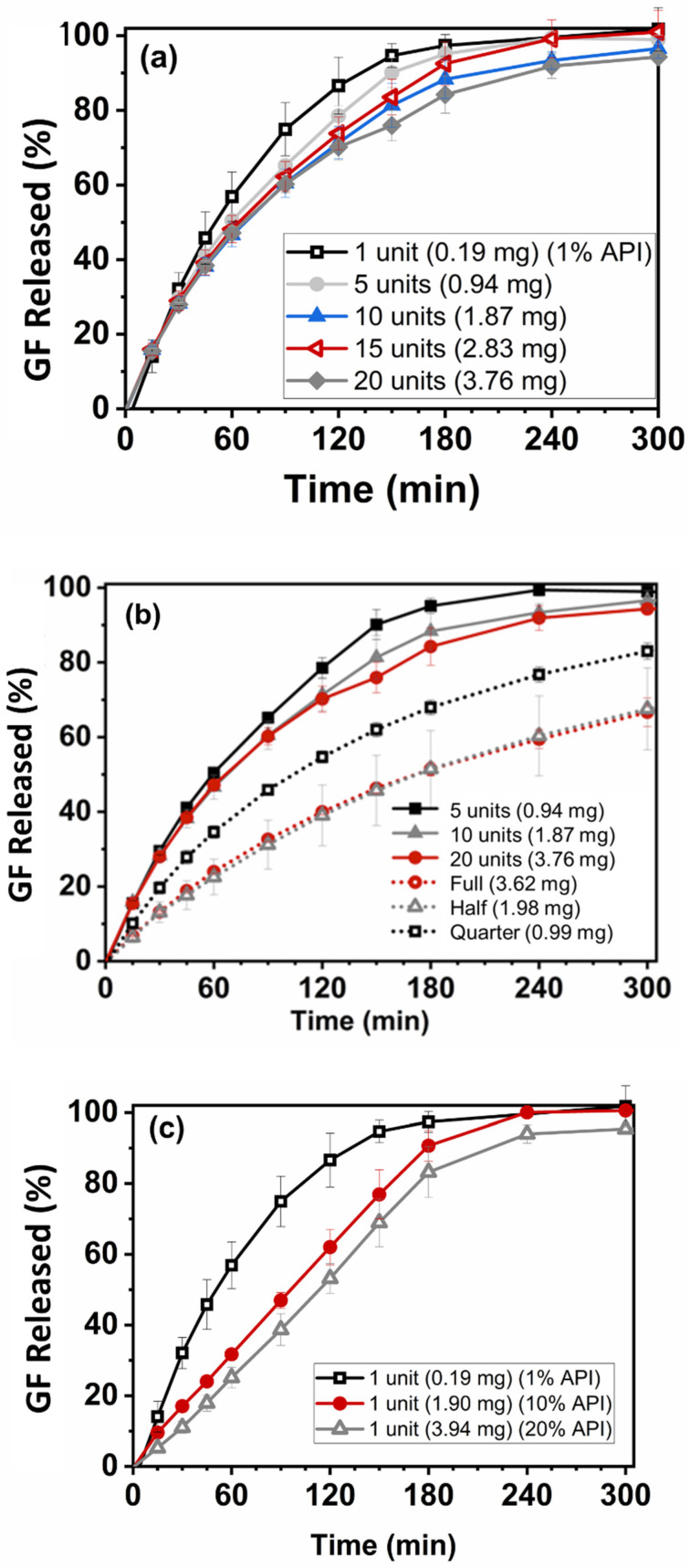
Drug release profiles of (**a**) single unit and multi units mini-tablet(s) at 1.0 wt% drug concentration, (**b**) split tablets at 1.0 wt% drug concentration, and (**c**) single unit mini-tablets at 1.0–20.0 wt% drug concentrations.

The release profiles of the 1–20 unit(s) mini-tablets are presented in Figure 5a, where the profiles of the composite units in ranges of 5–20 counts were found statistically similar with each other as per the bootstrap ƒ2 similarity test (see Table S2, Supplementary Material). However, the release profile of the single unit mini-tablet showed slight differences compared to that of multi-units. This could be the result of the low drug amount, ~190 µg, in a single tablet, which possibly caused variations in the individual release profiles, indicative of their release curve having relatively large standard deviations, see Figure 5a. Interestingly, Mitra et al. [21] suggested the need for additional method developments for testing the dissolution profile of a single mini-tablet at a low drug concentration. Nevertheless, these outcomes reinforced that using solidified HPC and the inherently dense matrix of FDM 3D-printed tablets [73,90,91] facilitated achieving similar release profiles from composite unit mini-tablets. As previously demonstrated [75], the resulting negligible variation in tablet size (see Table S1, Supplementary Material), implying closely similar SA/V for each individual mini-tablet, might have further contributed to the similarity in release profiles.

Incomplete dissolution profiles are commonly reported for 3D-printed tablets mostly due to their inherently dense matrix structure that limits water penetration in the dosage form [32,92]. On the contrary, nearly complete drug release was observed for the mini-tablets in this work, most likely due to their small size promoting the diffusion of the drug from its matrix. Palekar et al. [32] reported a similar observation, where enhanced drug diffusion was attributed to the smaller size of the 3D-printed mini-caplets because of the increased erosion of the polymeric matrix. Further, 3D printing provides great convenience for dose tailoring due to the flexibility with size, shape, and design of the final product [93,94]. These flexibilities offered by 3D printing can potentially affect the drug release behaviors [65,94]. Buyukgoz et al. [75] addressed this issue by printing fixed-size duo-tablets with internal placebo regions of varying sizes to achieve similar release profiles from the resulting constant surface area to volume ratio. Unfortunately, the duo-tablet dosage form is much larger in size and cannot offer as precise dose adjustment as the mini-tablets.

Next, the drug amounts were kept similar for comparing the split tablets and multi-unit tablets (see Table 3 for the details of the corresponding doses). The drug release profiles from printed split tablets are presented in Figure 5b. The release profiles of full, half-split, and quarter-split tablets were found to be statistically different according to the bootstrap ƒ2 similarity test, (Table S2, Supplementary Material). The full and half-split size tablets exhibited seemingly similar release profiles; however, large standard deviations appeared in the dissolution curve of the half-split tablets, most likely the reason of statistical difference. The release profiles of full, half, and quarter tablets exhibited slower drug release due to their significantly larger size over mini-tablets; therefore, the split-tablets could not compete with the performance of drug release rate and the consistency in the drug release from mini-tablets.

The release profiles of the mini-tablets at higher drug concentrations are presented in Figure 5c. The mini-tablets with 10.0 and 20.0 wt% drug concentrations showed statistically similar drug release profiles, yet different from the release profiles with the mini-tablets at 1.0 wt% drug concentration. This was expected since the large difference in the drug concentration range could change the drug release characteristic, although inherently dense FDM 3D-printed tablets [75,91] show some dominating effect on drug release. Nevertheless, GF concentration within the range of 10.0–20.0 wt% could help reduce the number of mini-tablets for the prescribed dose while providing similar drug release profiles.

Overall, this study assessed various design options for dose titration via FDM 3D printing by considering dose accuracy and consistency in drug release. As a major novelty, single unit mini-tablets enabled dose titration in steps of 0.19 mg with an enhanced content uniformity. Moreover, the composite unit mini-tablets showed similar release profiles. These results compare favorably with those offering dose adjustment by manipulating the tablet sizes, structure, or shapes via FDM 3D printing yet achieving different release profiles [62,95]. Thereby, this study demonstrates the feasibility of dispensing precise dosages with lesser manufacturing steps in mini-tablet manufacturing.

## 4. Conclusions

Tailored dosages were achieved via FDM 3D-printed mini-tablets by addressing the need for specific patient adjustment or titration in their prescribed dose. It was found that HME processing followed by FDM 3D printing promoted content uniformity, even for miniature tablets containing very low doses, and their label claim (LC) values were within the acceptable range. Such capability offered the desired flexibility in the range of 0.19–3.91 mg and high accuracy (R^2^ of 0.999) for precise dose titration through multi-unit mini-tablets. Further, reliable dosing was reinforced with similar drug release profiles from multi-unit mini-tablets afforded by the similarity in size and inherently dense structure. In contrast, the split tablets demonstrated different release behaviors, which was expected due to their size and shape differences. Additionally, mini-tablets with higher drug concentrations demonstrated similar release behaviors to each other. This capability points to the possibility of formulating the required dosage using mini-tablets at higher drug concentrations while reducing the number of tablets to be administered. The proposed approach to utilize FDM 3D printing to create mini-tablets with consistent drug release profiles eliminates many of the challenges associated with traditional tablet manufacturing while delivering high flexibility for drug delivery.

## Figures and Tables

**Table 1:** Compositions of the filament formulations.

Run	Formulation	HME Processing Temperature (°C)	HME Screw Speed (rpm)
F1	1 wt% GF + 84 wt% HPC + 15 wt% KP	150	40
F10	10 wt% GF + 75 wt% HPC + 15 wt% KP	150	40
F20	20 wt% GF + 65 wt% HPC + 15 wt% KP	155	40

**Table 2:** Tablet size and number of unit(s) of the printed mini-tablets.

Run		Theoretical Drug Concentration (wt%)	Radius * (mm)	Height * (mm)	Number of Unit(s)
M1		1% (F1)	1.5	2	1
M2			1.5	2	5
M3			1.5	2	10
M4			1.5	2	15
M5			1.5	2	20
M6		10% (F10)	1.5	2	1
M7		20% (F20)	1.5	2	1
F	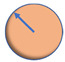		6.5	2.2	1
H	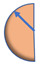	1% (F1)	6.5	2.2	1
Q	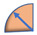		6.5	2.2	1

M: mini-tablet, F: full cylindrical, H: half of F, Q: quarter of F. The blue arrow in each tablet cartoon depicts its radius, see column 3 for the value. * Theoretical tablet dimensions.

**Table 3:** Drug dose titration for multi-unit mini-tablets and split tablets both at 1 wt% drug concentration, and for single unit mini-tablets at 1, 10, and 20 wt% drug concentrations.

Run	DC ^1^ (wt%)	Number of Tablet Unit(s)	Tablet Mass (mg)	Drug Mass (mg)	RSD	LC% ^2^	AV ^3^
M1	1	1	19.18 ± 0.32	0.19 ± 0.01	4.57	99.67 ± 3.92	7.83
M2	1	5	97.97 ± 1.95	1.04 ± 0.03	2.62	105.96 ± 0.84	0.44
M3	1	10	191.53 ± 1.12	1.93 ± 0.01	0.77	100.79 ± 0.22	1.78
M4	1	15	279.77 ± 3.84	2.85 ± 0.06	2.01	101.97 ± 0.66	2.39
M5	1	20	379.13 ± 2.15	3.91 ± 0.02	0.60	103.17 ± 0.36	6.49
F	1	1	312.23 ± 11.11	2.93 ± 0.07	2.44	93.92 ± 1.04	6.67
H	1	0.5	201.97 ± 2.73	1.95 ± 0.02	0.97	96.73 ± 0.50	2.77
Q	1	0.25	104.63 ± 0.73	1.00 ± 0.00	0.33	95.92 ± 0.08	2.75
M6	10	1	20.03 ± 0.39	1.95 ± 0.04	1.90	97.25 ± 0.52	2.30
M7	20	1	19.60 ± 0.32	3.76 ± 0.06	1.61	96.00 ± 0.35	3.19

^1^ drug concentration, ^2^ label claim %, ^3^ acceptance value.

## Data Availability

Additional data related to the paper is available and may be requested from the corresponding author.

## Supplementary Materials

The following supporting information can be downloaded at: https://www.mdpi.com/article/10.3390/pharmaceutics14112305/s1, Figure S1: The layered structure of mini-tablets at (a) 1 wt% and (b) 20 wt% drug concentrations; Figure S2: DSC thermograms of physical mixtures (PMs), GF-loaded filaments (FL) and the printed mini-tablets (3D) at 1 wt%, 10 wt%, and 20 wt% drug concentrations; Table S1: Drug content uniformity for single unit mini-tablet containing 1 wt% drug concentration; Table S2: Similarity (ƒ2) analysis for dissolution profiles of the 3D-printed tablets. References [96,97,98,99] are cited in the supplementary materials.
