# Supplementary material for: Dose Titration of Solid Dosage Forms via FDM 3D-Printed Mini-Tablets
Source: Pharmaceutics. 2022 Oct 27;14(11):2305. doi: 10.3390/pharmaceutics14112305 (PMC9695869; doi:10.3390/pharmaceutics14112305)
Supplement: Supplementary file 1 [file pharmaceutics-14-02305-s001.zip › pharmaceutics-1914759-SI.pdf]

Supplementary Material

Dose Titration of Solid Dosage Forms via FDM 3D-Printed Mini-Tablets

Guluzar Gorkem Buyukgoz [1,2], Christopher G. Kossor [1,2], Shen Ji[2], Murat Guvendiren[2] and Rajesh N. Davé [1,2,*]

[1] New Jersey Center for Engineered Particulates (NJCEP), New Jersey Institute of Technology, Newark, NJ 07102, USA;

[2] Otto H. York Department of Chemical and Materials Engineering, New Jersey Institute of Technology, Newark, NJ 07102, USA

* Correspondence: dave@njit.edu (R.N.D.); Tel.: +1 973-596-5860

Tablet morphology

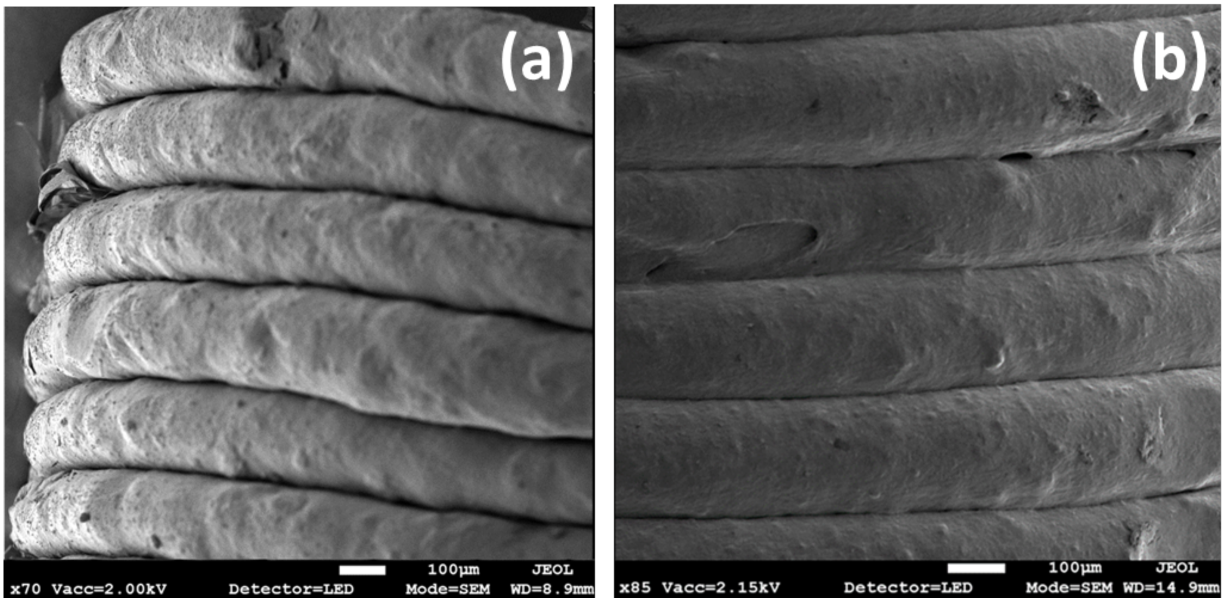

Figure S1. The layered-structure of mini-tablets at (a) 1 wt% and (b) 20 wt% drug concentrations.

Solid state changes

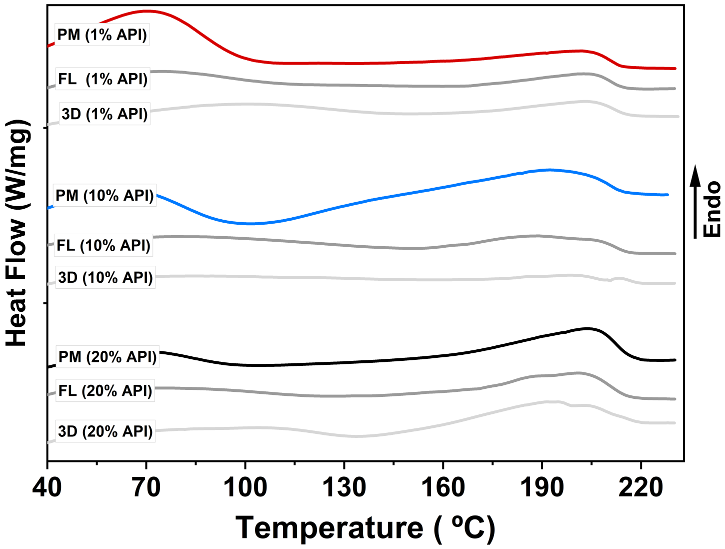

Figure S2. DSC thermograms of physical mixtures (PMs), GF loaded filaments (FL) and the printed mini-tablets (3D) at 1 wt%, 10 wt%, and 20 wt% drug concentrations.

PM DSC at 1 wt% showed broad endotherm. This is expected for PMs composed of a ternary blend when the components in the formulation have thermal events in a similar temperature range. For the HPC-KP mixture, it has been reported that although they are XRD amorphous [1,2] their PM (HPC-KP-1.0:7.5) showed melting endotherms around 166-218 ºC due to their small crystalline domains [3]. Since GF has a melting endotherm at ~220.4 ºC [4], similar thermal events in the similar temperature range were overlapped. This issue along with the low concentration of GF in 1 wt% formulations prevented a solid conclusion about the solid-state of GF. Similarly, a broad endotherm appeared in the PMs at 10 and 20 wt%. However, two endothermic shoulders were observed in the filaments and the printed tablets. Since HPC is commonly reported to cause melting point depression [3], the endothermic events could be attributed to the melting event of GF. These outcomes along with the XRD results may refer to the partial crystallinity of GF in filaments and the printed tablets at 10 and 20 wt%.

Dose titration

Table S1. Drug content uniformity for single unit mini-tablet containing 1 wt% drug concentration.

**Tablet mass (mg)**	**Diameter (mm)**	**Thickness (mm)**	**Drug mass (mg)**	**RSD**	**LC%**	**AV**
19.18±0.32	3.38±0.05	1.97±0.03	0.19±0.01	4.57	99.67±3.92	7.83

Similarity and difference factors for dissolution profiles of the 3D printed tablets

The dissolution profiles of the printed tablets in Table 3 were compared using bootstrap similarity (f_2) test [5,6] using PhEq_bootstrap software [5]. This test considers the variations between the individual dissolution profiles. For each case, the time point data beyond 85%

dissolution were discarded and the following bootstrapping parameters were applied; number of bootstraps are 5000, and confidence interval (CI) set to 90%. The 4 samples for each individual sub cases were used. The assessment of the results is based on the rule of dissolution profile similarity, where f_2 >50. The similarity statistics results are presented in Table S2.

Table S2. Similarity (f_2) analysis for dissolution profiles of the 3D printed tablets.

Run	Differences in Compared Couples			Similarity Factor (f_2)
	**Number of Unit(s)**	**Drug Concentration (%wt)**	**Tablet Size**	
1	1-5	1.0-1.0	M-M	53.63
2	1-10			45.68
3	1-15			47.89
4	1-20			45.64
5	5-10			59.06
6	5-15			59.04
7	5-20			55.22
8	10-15			68.42
9	10-20			67.44
10	15-20			64.04
11	1-0.5	1.0-1.0	F-H	28.66
12	1-0.25		F-Q	26.77
13	0.5-0.25		H-Q	40.67
14	1-1	1.0-10.0	M-M	32.79
15		1.0-20.0		27.45
16		10.0-20.0		52.94

M: Mini-tablet, F: Full size H: Half size Q: Quarter size tablet

References

1. Petry, I.; Löbmann, K.; Grohganz, H.; Rades, T.; Leopold, C.S. Solid state properties and drug release behavior of co-amorphous indomethacin-arginine tablets coated with Kollicoat® Protect. *European Journal of Pharmaceutics and Biopharmaceutics* **2017**, *119*, 150-160.
2. Rahman, M.; Coelho, A.; Tarabokija, J.; Ahmad, S.; Radgman, K.; Bilgili, E. Synergistic and Antagonistic Effects of Various Amphiphilic Polymer Combinations in Enhancing Griseofulvin Release from Ternary Amorphous Solid Dispersions. *European Journal of Pharmaceutical Sciences* **2020**, 105354.
3. Sarode, A.; Wang, P.; Cote, C.; Worthen, D.R. Low-viscosity hydroxypropylcellulose (HPC) grades SL and SSL: versatile pharmaceutical polymers for dissolution

enhancement, controlled release, and pharmaceutical processing. *Aaps Pharmscitech* **2013**, *14*, 151-159.
4. Buyukgoz, G.G.; Kossor, C.G.; Davé, R.N. Enhanced Supersaturation via Fusion-Assisted Amorphization during FDM 3D Printing of Crystalline Poorly Soluble Drug Loaded Filaments. *Pharmaceutics* **2021**, *13*, 1857.
5. Mendyk, A.; Pacławski, A.; Szlek, J.; Jachowicz, R. PhEq_bootstrap: open-source software for the simulation of f2 distribution in cases of large variability in dissolution profiles. *Dissolution Technology* **2013**, *20*, 13-17.
6. Paixão, P.; Gouveia, L.F.; Silva, N.; Morais, J.A. Evaluation of dissolution profile similarity–Comparison between the f2, the multivariate statistical distance and the f2 bootstrapping methods. *Eur. J. Pharm. Biopharm.* **2017**, *112*, 67-74.
